# The Impact of Bridging Additives on Wellbore Strengthening in Shallow Unconsolidated Formations

**DOI:** 10.1111/gwat.13455

**Published:** 2024-12-19

**Authors:** Alexis Koulidis, Tessel M. Grubben, Martin L. van der Schans, Martin Bloemendal, Philip J. Vardon

**Affiliations:** ^1^ Faculty of Civil Engineering and Geosciences Delft University of Technology Delft The Netherlands; ^2^ KWR Water Research Institute Nieuwegein The Netherlands; ^3^ TNO Geological Survey of the Netherlands Utrecht The Netherlands

## Abstract

Drilling wells in unconsolidated formations is commonly undertaken to extract drinking water and other applications, such as aquifer thermal energy storage (ATES). To increase the efficiency of an ATES system, the drilling campaigns are targeting greater depths and enlarging the wellbore diameter in the production section to enhance the flow rates. In these cases, wells are more susceptible to collapse. Drilling fluids for shallow formations often have little strengthening properties and, due to single‐string well design, come into contact with both the aquifer and the overburden. Drilling fluids and additives are experimentally investigated to be used to improve wellbore stability in conditions simulating field conditions in unconsolidated aquifers with a hydraulic conductivity of around 10 m/d. The impact on wellbore stability is evaluated using a new experimental setup in which the filtration rate is measured, followed by the use of a fall cone penetrometer augmented with an accelerometer to directly test the wellbore strengthening, and imaging with a scanning electron microscope (SEM) to investigate the (micro)structure of the filter cakes produced. Twelve drilling fluids are investigated with different concentrations of bentonite, polyanionic cellulose (PAC), Xanthan Gum, calcium carbonate (CaCO_3_), and aluminum chloride hexahydrate ([Al(H_2_O)_6_]Cl_3_). The filtration results indicate that calcium carbonate, average *d*_*p*_ <20 *μ*m, provides pore throat bridging and filter cake formation after approximately 2 min, compared to almost instantaneous discharge when using conventional drilling fluids. The drilling fluid containing 2% [Al(H_2_O)_6_]Cl_3_ forms a thick (4 mm) yet permeable filter cake, resulting in high filtration losses. The fall cone results show a decrease of cone penetration depth up to 20.78%, and a 40.27% increase in deceleration time while penetrating the sample with CaCO_3_ compared with conventional drilling fluid containing bentonite and PAC, indicating a significant strengthening effect. The drilling fluids that contain CaCO_3_, therefore, show high promise for field implementation.

## Introduction

The number of groundwater wells has grown dramatically over the years, with an estimated 2 million wells drilled every year globally (NWP 2007). In the Netherlands, around 75% of the groundwater wells are utilized to produce water (Schuerhoff and Hellegers 2015), with other uses including the storage of thermal energy. Generally, excess (sustainable) thermal energy is available in the summer, while there is insufficient supply in the winter. Utilizing groundwater wells to access aquifers, seasonal storage of thermal energy is known as aquifer thermal energy storage (ATES). ATES systems have been shown to be reliable thermal energy storage systems (Bloemendal and Hartog 2018; Oesterholt et al. 2018; Bloemendal et al. 2021), which leads to a growth in drilling groundwater wells for this technology. Currently, more than 3000 ATES systems are installed in the Netherlands (Bloemendal et al. 2022).

With the growth of the installation of groundwater wells, less permeable aquifers are targeted, which makes drilling operations, well development, and cost mitigation more challenging (Perrone and Jasechko 2019). Challenges with optimizing well performance occur during drilling and completion, especially when targeting more challenging aquifers. Major factors that influence the groundwater well's injectivity and productivity include (1) the natural hydraulic conductivity of the aquifer (Sanchez‐Vila et al. 2006), (2) the skin formed in the wellbore (produced by the drilling fluid) (van Lopik 2020), and (3) completion design, which relates to the wellbore diameter (Houben 2015). This leads to a desired increase of borehole diameters to enhance well capacity (van der Schans et al. 2022). In a study by van der Schans et al. (2022), the borehole diameter was artificially increased from 600 mm to approximately 1500 mm. The results showed that the larger borehole surface area increased the design flow rate by a factor of 2 compared with conventional reference wells.

The target aquifers in the Netherlands are unconsolidated and permeable formations, with a hydraulic conductivity ranging from 10 to 50 m/d (de Vries 2007; van der Schans et al. 2022), which induce excessive head losses, wellbore instability, and low productivity (poor flow). These aquifers are often relatively shallow, for example, a few hundred meters, which significantly limits the chemicals allowed to be used during drilling or well development (Brobst and Buszka 1986; Regenspurg et al. 2018), for example, to protect (shallower) drinking water resources from bacterial growth. Environmental regulations and drilling rig mechanical capacity require a drilling fluid that, from a rheological perspective, acts like a standard fluid but also bridges the pore throats of the formation reducing permeability. Thus, a key challenge is to design and select an environmentally friendly drilling fluid that enhances wellbore strength and allows for the initial permeability to be restored after well development. Thus, well development is a principal aspect of those wells and has to be considered during well planning. A common practice for drilling wells in the Netherlands is to use reverse circulation. This drilling technique imposes several limitations with regard to the rheological properties of the drilling fluid that is circulated in the wellbore (common field practice is a maximum value of plastic viscosity of 9 cP). In this method, the fluid in the annulus is driven downwards purely by gravity and enters the drill head. Subsequently, the fluid is lifted through the drill pipe by air injection. Water‐based drilling fluids are typically used since the wells are shallow and close to potable water resources.

**Figure 1:**
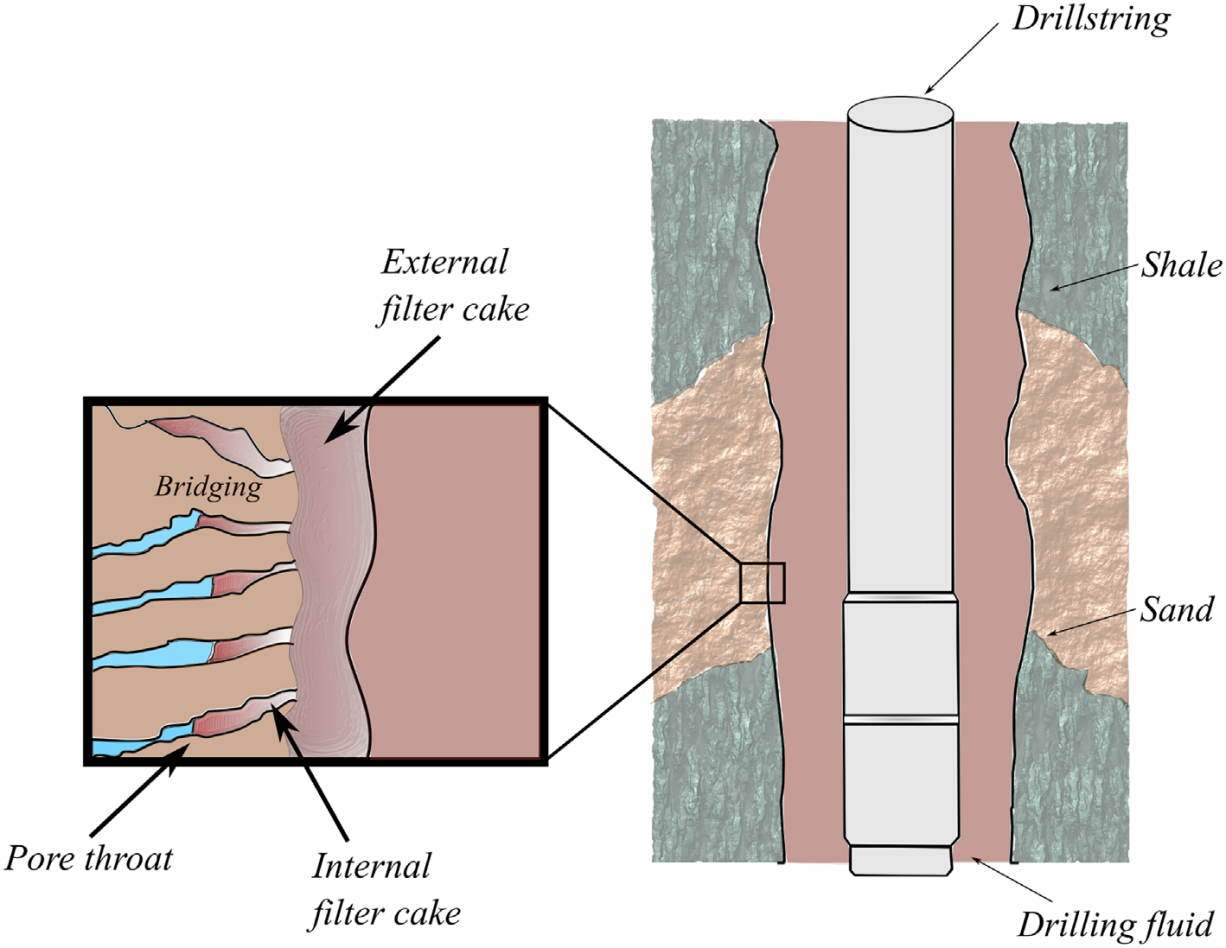
Schematic of filter cake development while drilling permeable formation.

Unconsolidated formations often have layers of low cohesion and strength, which create challenges while drilling (Zhao et al. 2019; Zhang et al. 2022; Zhao et al. 2022). During the drilling process, a drilling fluid is circulated from the surface to the drill bit through the drill string/pipe and returned through the annulus or vice versa. The drilling fluid serves a number of functions, including transporting cuttings out of the well, reducing or eliminating flow into the well, and the preservation of wellbore stability (Skenderija et al. 2021; Skenderija et al. 2024). The performance of the drilling fluid depends on several variables, including its viscosity which assists in cuttings transport, the ability to partially support the borehole, and reducing drilling fluid infiltration into the formation. To prevent the formation fluids from entering the wellbore, the fluid pressure provided by the drilling fluid should be higher than the pore pressure. This difference creates a tendency for the fluid to flow into (infiltrate) the formation and the solid particles in the drilling fluid to concentrate at the wellbore wall due to the filtration effects of the formation (Yao et al. 2014; Feng et al. 2018; Elkatatny 2019). The filtration of the drilling fluid into the formation can be divided into several phases. Initially, the fluid comes in contact with a clean wellbore wall, and the early stage filtration occurs at the borehole surface (Ezeakacha et al. 2017; Zhang et al. 2022). In the second stage, as the solids accumulate on the wellbore surface, particles invade the pores in the near wellbore region with the particles (partially) bridging the pore throats (Figure 1), forming an internal filter cake. The bridging changes the effective stress locally and reduces the near wellbore permeability (Magzoub et al. 2021). In the third stage, an external filter cake starts developing as the internal filter cake prevents more solids from entering the pore network.

The filter cake has a number of positive and negative effects on the overall drilling process. First of all, an impermeable filter cake reduces the filtration rate into the formation, which increases the pressure on the wellbore, increasing stability and providing direct wellbore strengthening (Klungtvedt et al. 2023). On the other hand, an impermeable filter cake can reduce the well's productivity unless it can be cleaned. Ideally, a filter cake is created and present during drilling and is easily removable prior to use. Thus, an impermeable and thin filter cake is desired for ATES and groundwater wells.

Filter cake development is a function of fluid properties, the differential pressure between the wellbore and formation pressure, formation permeability and porosity, time, and temperature (Cerasi et al. 2001; Ma et al. 2020). For a low‐quality filter cake (high filtration rate and large thickness), the fluid can easily flow into the formation and locally increase the pore pressure, which corresponds to a reduction of the circumferential stress (hoop stress) (Feng et al. 2018). Significant reduction of the hoop stress, exceeding the tensile strength, results in wellbore collapse (Bagheri et al. 2021). A major challenge for drilling large‐diameter wells in permeable unconsolidated formations is the significant fluid losses corresponding to a reduction in wellbore pressure. Previous experience with large‐diameter wells showed that relatively small reductions in fluid level may result in wellbore collapse (van der Schans et al. 2022). Therefore, ideally, the drilling fluid forms a filter cake in a short period of time.

To form the filter cake, a bridging mechanism has to be accomplished (Cook et al. 2016; Ezeakacha et al. 2017; Klungtvedt et al. 2023). This is often achieved by adding additives to the drilling fluid. Table 1 summarizes water‐based drilling fluid additives that assist wellbore strengthening by reducing filtration rates. The drilling fluid composition and materials are selected for each well based on formation characterization and other constraints, for example, environmental requirements. Formation properties, including permeability, pore throat diameter, uniformity, and porosity, assist in selecting the optimum additives in the drilling fluid (Ezeakacha et al. 2017). Several researchers have shown that the particle size distribution (PSD) of the drilling fluid additives plays an important role in the formulation of the filter cake (Ma et al. 2020; Klungtvedt and Saasen 2023). Conventional API filtration tests do not give a practical output for unconsolidated formations since the PSD of specific aquifer sand has a major effect on the performance of the bridging material (Dehghani et al. 2019; Villada et al. 2022). For the evaluation of filtration losses in unconsolidated samples, multifunctional experimental setups have been designed (Amanullah and Boyle 2006; Zhao et al. 2019). However, a quantifiable measurement that is related to wellbore strengthening is currently lacking. A common and versatile application to assess near‐surface sediment shear strength is the free fall penetrometer (FFP) equipped with an accelerometer (Dayal 1980). As the cone penetrates the sediments, the cone resistance, acceleration/deceleration, and side friction are measured, with the sediment strength being directly proportional to the deceleration profile (Stark et al. 2014; Stark et al. 2022). At the lab scale, a similar test, that is, the fall cone penetrometer is used to determine the undrained shear strength. The concept is similar to the FFP, but it is not generally instrumented. A previous study by Zhao et al. (2019) showed that the needle penetration test is a reliable measurement for evaluating the effect of drilling fluid on wellbore strengthening. The results showed an increase in the compressive strength (3.23% to 17.26%) and a decrease in penetration depth (25% to 62.5%), while using 2 wt% Al‐seal.

**Table 1:** Drilling Fluid Additives Used to Form a Filter Cake

References	Additives	Results
Sami 2015	Gypsum	Lower thermal degradation at 200°F and better filtration properties compared to lignite drilling fluid
Zhao et al. 2019	Aluminum complex seal	Permeability reduction by 82.42% Penetration reduction by 62.5%
Dehghani et al. 2019	CaCO_3_ nanoparticles	Reduction of fluid loss volume and filter cake thickness by 26% and 64%, respectively
Elmgerbi et al. 2021	CaCO_3_, Mikhart 10 and Mikhart 65	Filtration volume does not have a relation with residual damage
Villada et al. 2022	CaCO_3_ (fine, medium, and coarse), graphite, and lignite	Shear stress and viscosity decreased with the increase of CaCO_3_ concentration %
Ali et al. 2022	Peel powder, SiO_2_, and TiO_2_ nanoparticles	Reduction of fluid loss volume by 31% and 25.8%, respectively

In this work, a framework to experimentally determine the drilling fluid performance for unconsolidated formations is established. A modified apparatus is designed to include the impact of the formation, which can be used to evaluate both internal and external filter cake development and assess the filtration losses and the strength of the near wellbore region.

## Experimental Procedure

The experimental procedure is divided into three segments: (1) material characterization; (2) rheological characterization; and (3) evaluation of the effect of drilling fluid on hydraulic conductivity and wellbore stability. The selection of the additives is made with the consideration of the well development process, which will be discussed.

### Materials

#### Drilling Fluids

For this study, drilling fluids have been designed with different concentrations of bentonite, Xanthan Gum, polyanionic cellulose (PAC), calcium carbonate (CaCO_3_), and aluminum chloride hexahydrate ([Al(H_2_O)_6_]Cl_3_). Bentonite functions as the main viscosifier and prevents fluid loss, with Xanthan Gum a secondary additive that performs similar functions. PAC is a filtration control agent, that is, forming a low permeability filter cake, and increases viscosity. CaCO_3_ and [Al(H_2_O)_6_]Cl_3_ can provide bridging between the soil particles. Table 2 describes the composition of the drilling fluids that have been tested. The additives and compositions are selected considering effective bridging properties from the literature and common additives used to drill water wells. Regarding the mixing procedure, initially, water is mixed with bentonite, followed in order, when used, by PAC, CaCO_3_/[Al(H_2_O)_6_]Cl_3_, and finally, Xanthan Gum. Each new additive is included at time intervals of 10 min to ensure a homogeneous mixture.

**Table 2:** Tested Drilling Fluids (wt%)

Additives	Abbreviations
6% bentonite	#1: 6B
2% bentonite and 0.05% PAC	#2: 2B‐0.05P
1% bentonite, 0.5% PAC, 0.5% Xanthan Gum, and 0.25% CaCO_3_	#3: 1B‐0.5P‐0.5X‐0.25Ca
1% bentonite, 0.5% PAC, 0.5% Xanthan Gum, and 1% CaCO_3_	#4: 1B‐0.5P‐0.5X‐1Ca
1% bentonite, 0.25% PAC, 0.25% Xanthan Gum, and 1% CaCO_3_	#5: 1B‐0.25P‐0.25X‐1Ca
1% bentonite, 0.1% PAC, 0.1% Xanthan Gum, and 1% CaCO_3_	#6: 1B‐0.1P‐0.1X‐1Ca
1% bentonite, 0.1% PAC, and 1% CaCO_3_	#7: 1B‐0.1P‐1Ca
1% bentonite, 0.2% PAC, and 1% CaCO_3_	#8: 1B‐0.2P‐1Ca
1% bentonite, 0.1% PAC, and 1% CaCO_3_	#9: 2B‐0.1P‐1Ca
1% bentonite, 0.5% PAC, and 2% Gypsum	#10: 2B‐0.5P‐2G
1% bentonite, 0.2% PAC, and 1% Aluminum chloride hexahydrate	#11: 1B‐0.2P‐1Al
2% bentonite, 0.1% PAC, and 2% Aluminum chloride hexahydrate	#12: 2B‐0.1P‐2Al

#### Soil Sample

**Figure 2:**
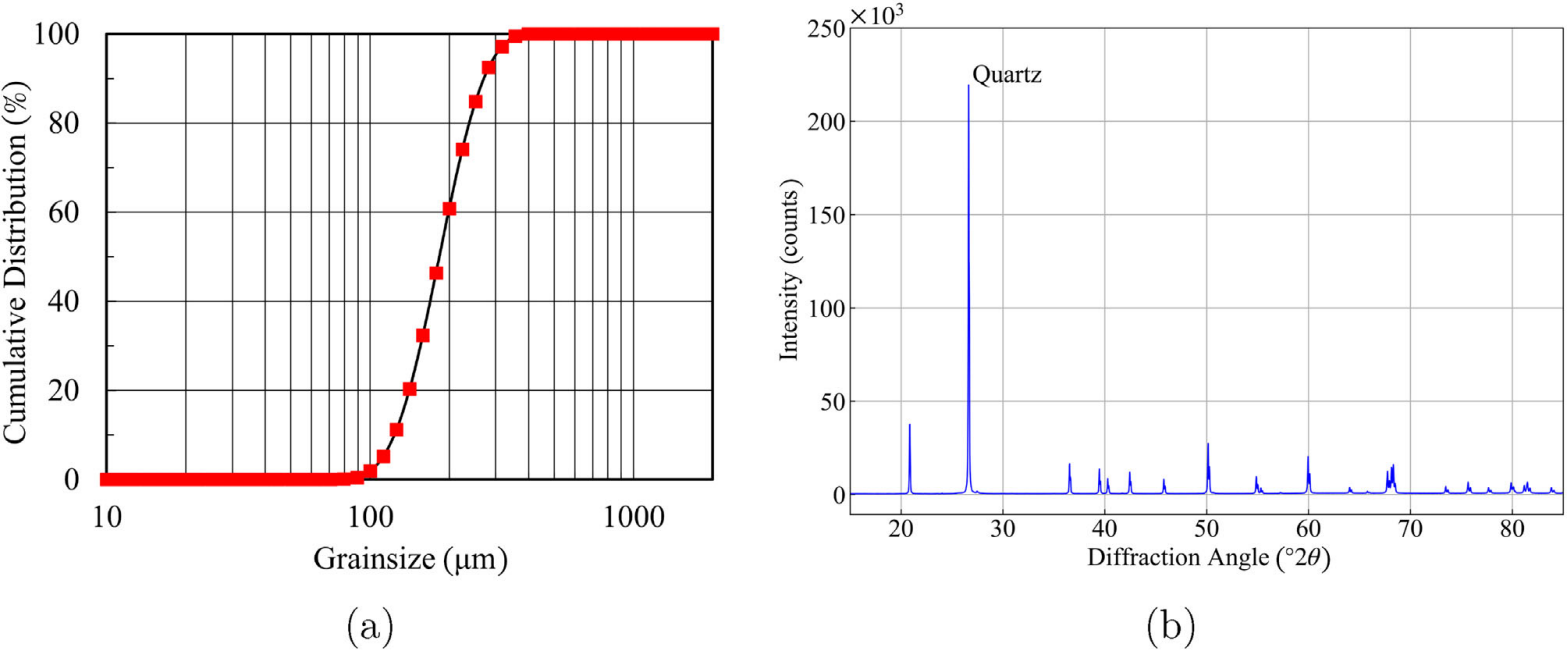
(a) Particle size distribution of the sand simulating the target formations; (b) X‐ray diffraction patterns for the tested sand sample.

Core samples are analyzed from an ATES exploration well in Delft, the Netherlands, to ensure representative formation properties. The sand sample selected to simulate the formation has particle diameters (*d*_*p*_) between 125 and 350 *μ*m and is expected to have a hydraulic conductivity of 10 m/d. The PSD of the selected sand is presented in Figure 2a. The mineralogical composition of the sand is determined by means of X‐ray diffraction (XRD) Figure 2b. Approximately 10 g of ground sand is scanned with a reflection angle (2*θ*) from 15 to 85° with a 1‐s time step. The mineralogical analysis shows quartz as the main mineral.

### Rheological Properties and Conventional Filtration (API Filter Press)

The rheological properties of the drilling fluids are determined using a Fann 35 viscometer. Constant measurements are obtained at speeds (*θ*) of *θ*600, *θ*300, *θ*200, *θ*100, *θ*6, and *θ*3. In addition, the gel strength is obtained after both 10 s and 10 min in static conditions. The plastic viscosity *μ*_*p*_ and the yield point *y*_*p*_ are calculated as:

(1) $$\mu_{p}=\theta 600-\theta 300\;\left(\mathrm{cP}\right)$$

(2) $$y_{p}=\theta 300-\mu_{p}\;\left(\mathrm{lb}/100\;{\text{ft}}^{2}\right)$$

Filter press experiments (API RP 13B‐1 2009) simulate the behavior of a drilling fluid under low temperature and pressure. The sample is placed in the cell and pressurized while the discharged fluid volume is measured at specific time intervals. The solid particles are deposited on a filter paper, with a filtration area of 7.1 in^2^ (45.8 cm^2^) and particle retention above 2.7 *μ*m. Due to the shallow depth that the unconsolidated formations encounter, the applied pressure is decreased from the standard 690 kPa (100 psi) to 200 kPa (29 psi), reflecting a more representative overbalance pressure. The total duration of one experimental run is 30 min, and the fluid loss is measured at 7.5 min intervals. After 30 min, the filter cake thickness and water content of the filter cake are measured.

### Modified Filtration Test

**Figure 3:**
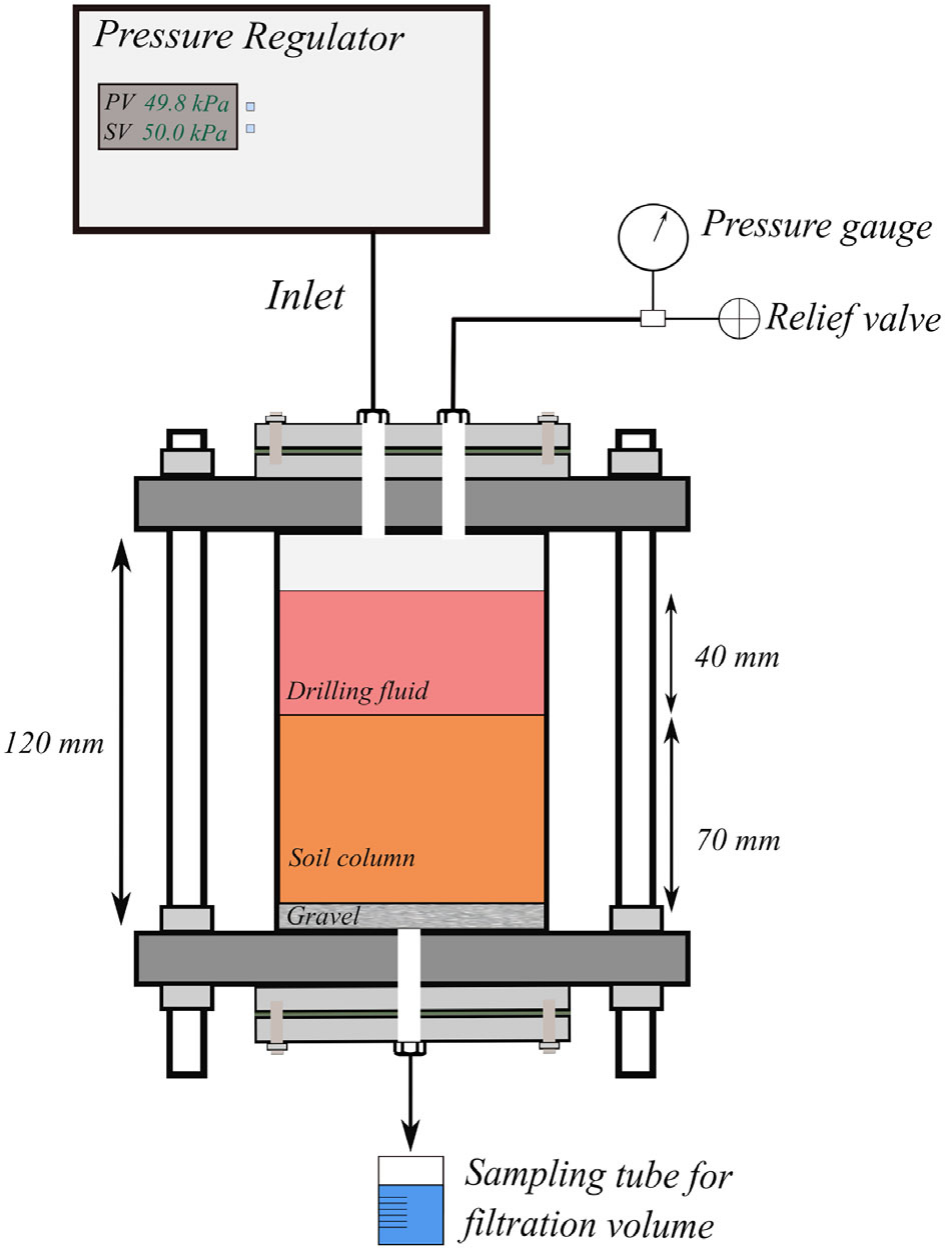
Modified filtration apparatus with the corresponding mechanical components. The upper and lower caps that are attached to the main frame allow simple and accessible assembly and disassembly of the apparatus.

The experimental setup is shown in Figure 3. The setup is based on a triaxial cell, of 12 cm length and 9.4 cm diameter. The volume of the apparatus is approximately 832 cm^3^ with a cross‐sectional (filtering) area of 69.36 cm^2^. The frame consists of stainless steel caps and supporting rods and a 5 mm thick plexiglass cylinder with several gaskets to act as a sealing mechanism between different parts in the upper and lower caps. The six vertical stainless steel rods allow for effective sealing of the upper and lower parts of the apparatus. The setup has been pressure tested to six bar.

The modified apparatus allows testing of filtration rates and the formation of filter cake for the pressure range of 50 to 690 kPa. In this paper, to represent the overbalanced pressure used in the typical drilling of shallow ATES wells, a pressure of 50 kPa is used. The applied pressure is lower than in the API filtration tests, that is, 50 and 200 kPa, respectively. Initially, experiments with the API filtration apparatus show that for the tested drilling fluids the 50 kPa applied pressure does not create sufficient thickness filter cake. A summary of the operating procedure is:

- The cell is filled with a uniform mixture of sand in layers of 1 cm and each layer is compacted with a tamper 15 to 20 times. The surface of each layer is scraped with a small trawl to ensure interlocking with the subsequent layer.
- The upper cap is fixed and the cell is fully assembled.
- Demineralized water is pumped inside the cell and pressurized to 50 kPa to saturate the formation.
- The discharge valve is opened and the hydraulic conductivity (*k*) is calculated.
- The drilling fluid is carefully poured on top (by opening the upper cap) of the saturated sample and the cell is again pressurized at 50 kPa.
- After 5 min, the discharge valve is opened and the filtration test is initiated. The total duration of each experiment is 1 h. Filtration losses are measured at 2.5 min intervals for 40 min, and then at 5 min intervals for the following 20 min.
- The remaining drilling fluid is removed carefully with a syringe. Subsequently, the upper part of the apparatus is disassembled.
- A cylindrical coring device is utilized to obtain an undisturbed core sample from the center of the apparatus with a diameter of 4 cm, which is used for the fall cone penetrometer test and the scanning electron microscope (SEM).

### SEM and Particle Size of Calcite Crystals

**Figure 4:**
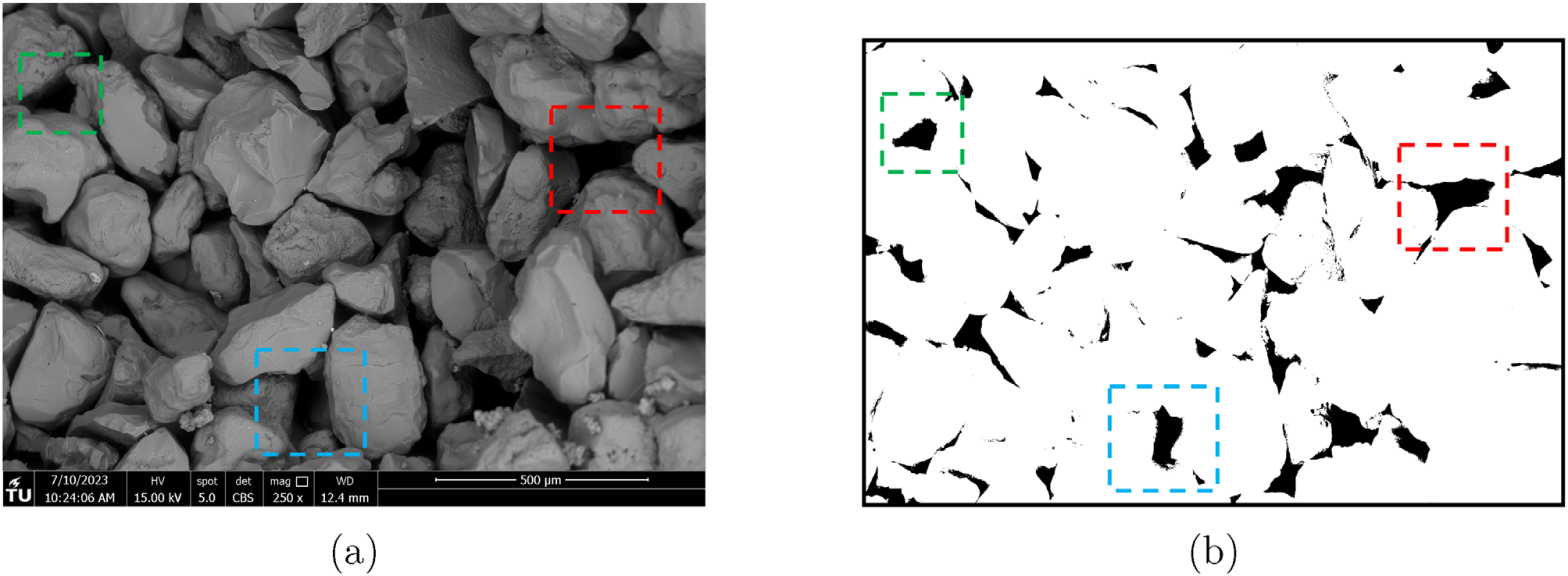
(a) Unprocessed image obtained from SEM illustrating the pores of the sand formation. (b) Binary segmented image after thresholding showing the pore areas (black) and the connections among the grains.

To evaluate the morphology and penetration depth of the filter cake, we conduct SEM imaging of the formed filter cake at the upper 2 cm of the sample. Image segmentation is performed using ImageJ (Schneider et al. 2012), following the method of Hojat et al. 2023. The original image contains a scale bar, which is utilized to convert the pixels to a length scale (Figure 4a).

**Figure 5:**
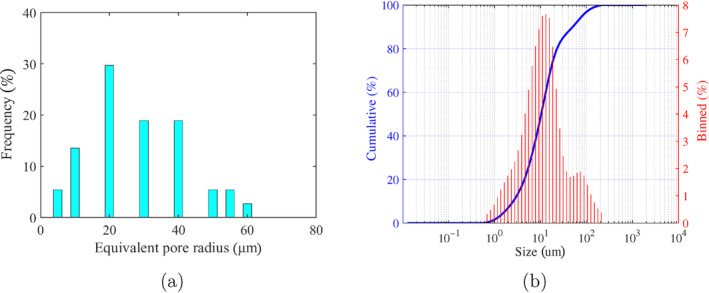
(a) The distribution of equivalent pore radius using SEM images; (b) Particle size distribution of calcite crystals.

The pores are identified by applying a threshold value and identifying the morphological features in the image as illustrated in (Figure 4b). The extracted area of each pore is then used to calculate the equivalent pore radius as $r_{\text{pore}}=\sqrt{A/\pi }$. It is observed in Figure 5a that the highest percentage of the pore size is below 40 *μ*m. Similarly, the initial porosity is estimated before the injection of the drilling fluids as approximately 30%.

The PSD of the calcite crystals is measured with a laser diffraction PSD analyzer as illustrated in Figure 5b. The optimum PSD significantly impacts the effect of pore throat bridging. However, for the current case study, we report only the PSD of the calcite crystals and not of the drilling fluid.

### Fall Cone Test with Mounted Accelerometer

**Figure 6:**
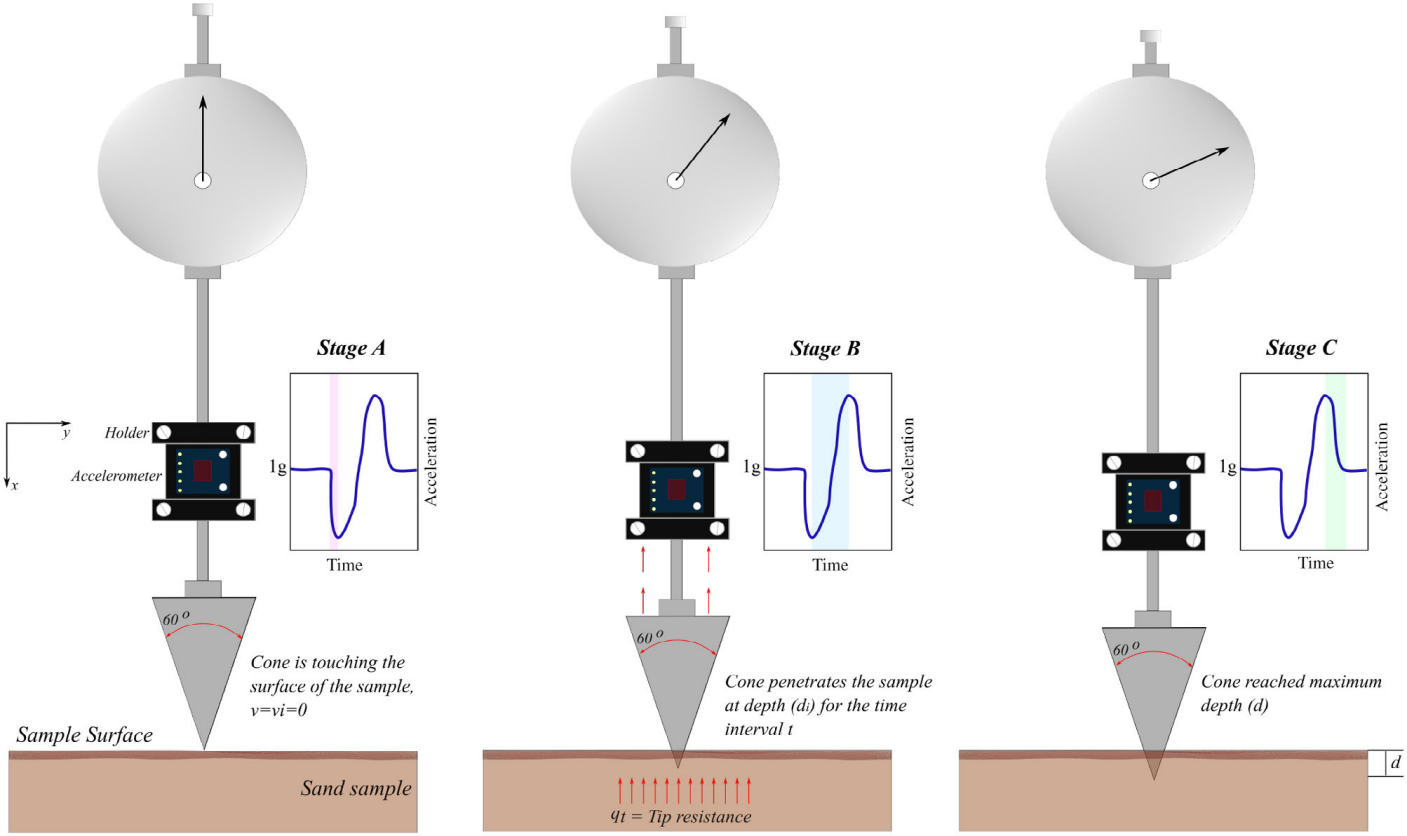
Modified cone penetrometer setup. Left: Stage A, Middle: Stage B, Right: Stage C.

The standard fall cone test measures only penetration depth, and does not give details of the deceleration motion. As the filter cake leads to a layered surface, a three‐axis accelerometer is mounted above the cone, which acquires high‐frequency (570 Hz) acceleration data during the penetration. The accelerometer is connected to an Arduino Uno, with the total additional weight in the apparatus being 15 g. As the target is the wellbore strengthening, the external filter cake is carefully removed to eliminate any additional acceleration that is not related to the cone penetration in the sample. As the cone penetrates the sample, it encounters the internal filter cake followed by the unmodified sand. As the cone penetrates the soil sample, it decelerates due to resisting forces. By analyzing the motion of the cone, the wellbore strengthening effect can be quantified. Dynamic acceleration data and cone penetration depth are taken for each analysis. Figure 6 shows the schematic of the modified fall cone test, which follows the stages below:

- *Stage A*: The cone is positioned directly above the sample, that is, nearly touching the surface of the sample (where the measured acceleration value is 1 g), before the penetrometer is released. The penetrometer is released and starts to penetrate the sample, until it reaches a maximum acceleration.
- *Stage B*: The penetrometer decelerates due to the resistance of the sample until the maximum deceleration (acc).
- *Stage C*: The penetrometer continues decelerating at a slower rate until it is again at rest and at its maximum penetration depth (*d*). The measured acceleration value is then again 1 g.

Prior to the drilling fluid tests, identical sand samples are tested in the cone penetrometer test with 0% water content (dry) and fully saturated with demineralized water to obtain benchmark values.

## Results

### Rheological Properties

**Figure 7:**
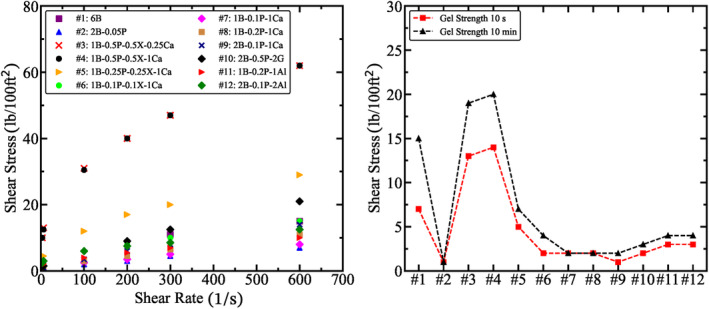
Rheological behavior of drilling fluids for different shear rates. Left: Shear stress against shear rate; Right: Gel strength.

**Figure 8:**
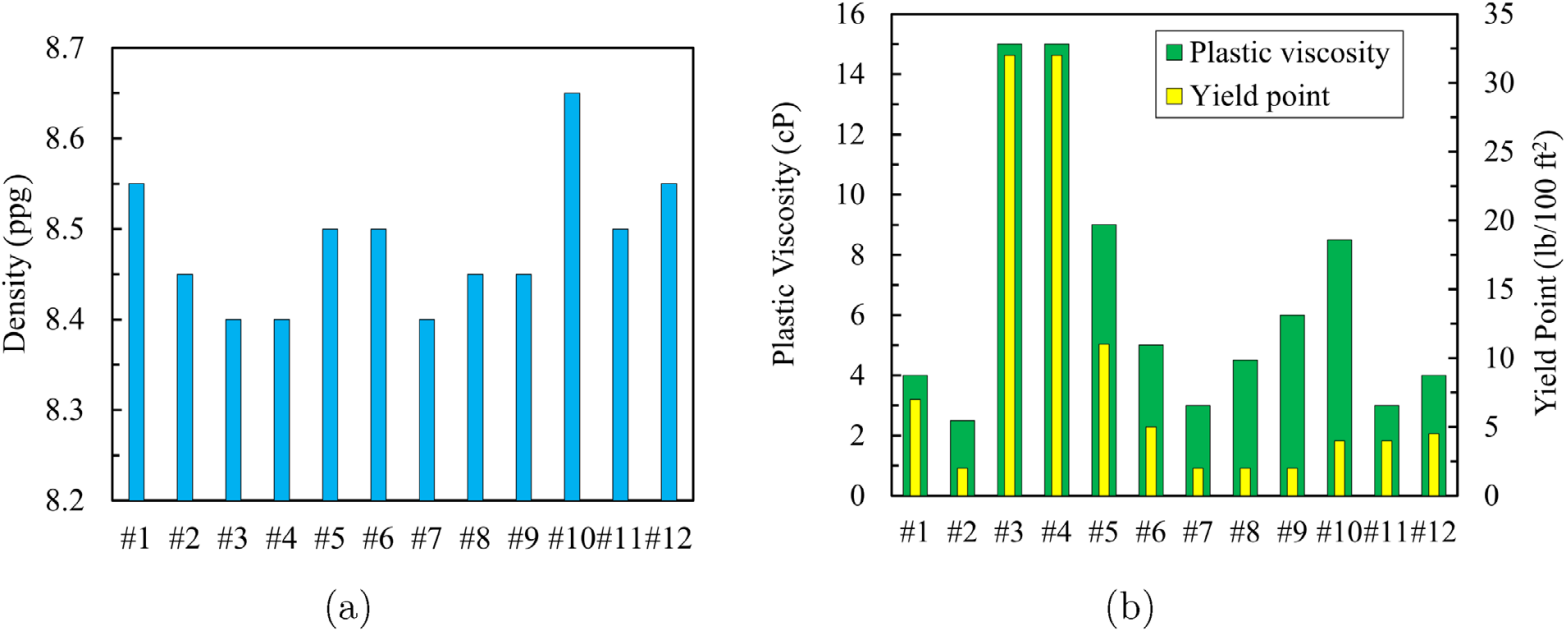
(a) Drilling fluid density for drilling fluids with different additives using mud balance; (b) Plastic viscosity and yield point.

Figure 7 shows the rheological behavior of the drilling fluids for different shear rates. From Figure 7, it is observed that an increase of CaCO_3_ in the sample (comparing results of #3 and #4) does not affect the rheological properties of the drilling fluid, probably since the quantities are relatively low. As the concentration of PAC and Xanthan Gum in the drilling fluid is increased, the fluid changes from behaving as a Bingham‐plastic, that is, having a linear relationship between shear stress with shear rate (#2, #6, #7, #9 to #12), to behaving as a Hershel‐Bulkley fluid, that is, having a non‐linear relationship of shear stress with shear rate (#3, to #5, #8). For drilling fluids #3 and #4, the gel strength shows a significant increase compared to all other drilling fluids. The drilling fluid density and plastic viscosity are presented in Figure 8. The results show that adding Xanthan Gum in significant quantities significantly influences the viscosity, not the density, whereas adding significant quantities of bentonite, calcite, aluminum chloride hexahydrate, and gypsum increases the density. The maximum permissible plastic viscosity is 9 cP.

### Filtration Tests

#### API Filter Press

**Figure 9:**
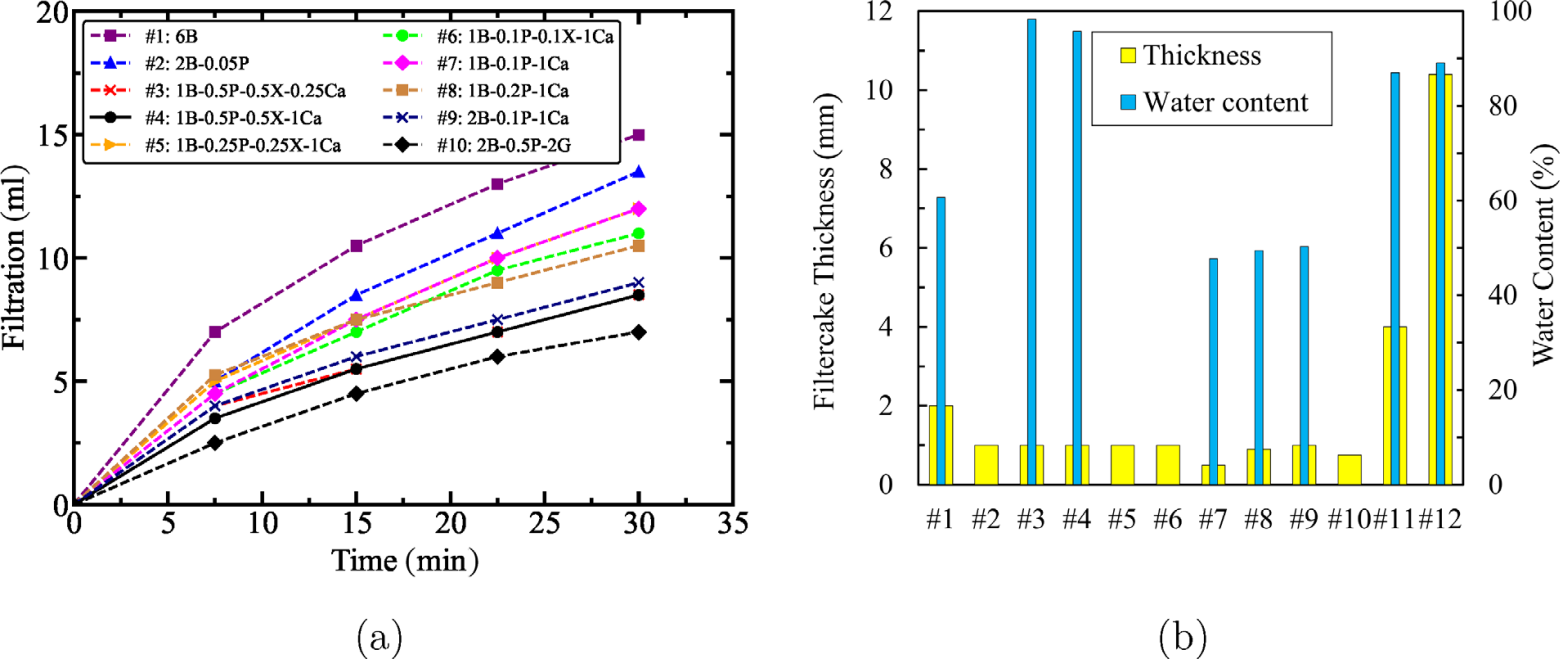
(a) Cumulative filtration during the API filter press test (results from drilling fluids #11 and #12 are not included due to their high rate); (b) Filter cake thickness and water content for different drilling fluids. Due to similar chemistry to other drilling fluids and relatively thin filter cake, the water samples from drilling fluids #2, #5, #6, and #10 were not acquired.

The results of the API filter press tests conducted for all the drilling fluids are presented in Figure 9a. Drilling fluids #11 and #12 have 293 and 263 mL total filtration at the 30‐min interval, respectively, and for that reason, are not shown in the figure. Of the remaining drilling fluids, the 6% bentonite drilling fluid (drilling fluid #1) shows the highest filtration rates. It can be observed that by adding 0.05% PAC and reducing the concentration of bentonite in the drilling fluid (drilling fluid #2), the filtration losses are reduced by 13%. Adding bridging or viscosity increasing agents can have a significant impact, reducing filtration losses by up to 50%.

The filter cake thickness and water content have been measured for the different drilling fluids, and are presented in Figure 9b. There is no direct relationship between filter cake thickness, water content, and filtration losses observed. For example, even though the drilling fluids #3 and #4 have significantly higher viscosity and lower filtration rates, their corresponding filter cake thickness is seen to be equal to that of drilling fluids #2, #5, and #6. In addition, drilling fluids #11 and #12, including aluminum chloride hexahydrate, form a thick, permeable filter cake with filtration loss on 7.5 min of 140 and 123 mL, respectively. The total water content of the filter cake sample is determined by measuring the weight before and after drying it in the oven for 12 h. The chemical composition of drilling fluid #10 slightly differs since gypsum is the main additive. The relatively thin filter cake creates an additional challenge to acquire a small sample to measure the water content.

#### Modified Filtration Test

The unconsolidated sand samples are prepared with characteristics similar to the composition and particle size of the formation of interest. As discussed, the samples are not cemented but compacted, and it is observed that during the initial saturation, a slight uplift is observed in the upper section compared to the lowest part of the sample. Even though the same procedure and injection flow rate are followed for all the experiments, a difference in the compaction rate in some of the samples is observed. During the saturation process, initially, the fluid follows the most accessible pore path and is followed by saturating the entire sample for a specific height. Due to this phenomenon, the standard deviation of the measured hydraulic conductivity of every sample is equal to 0.62 m/d and an average value of 10.84 m/d, which demonstrates the reliability and repeatability of the experiments.

**Figure 10:**
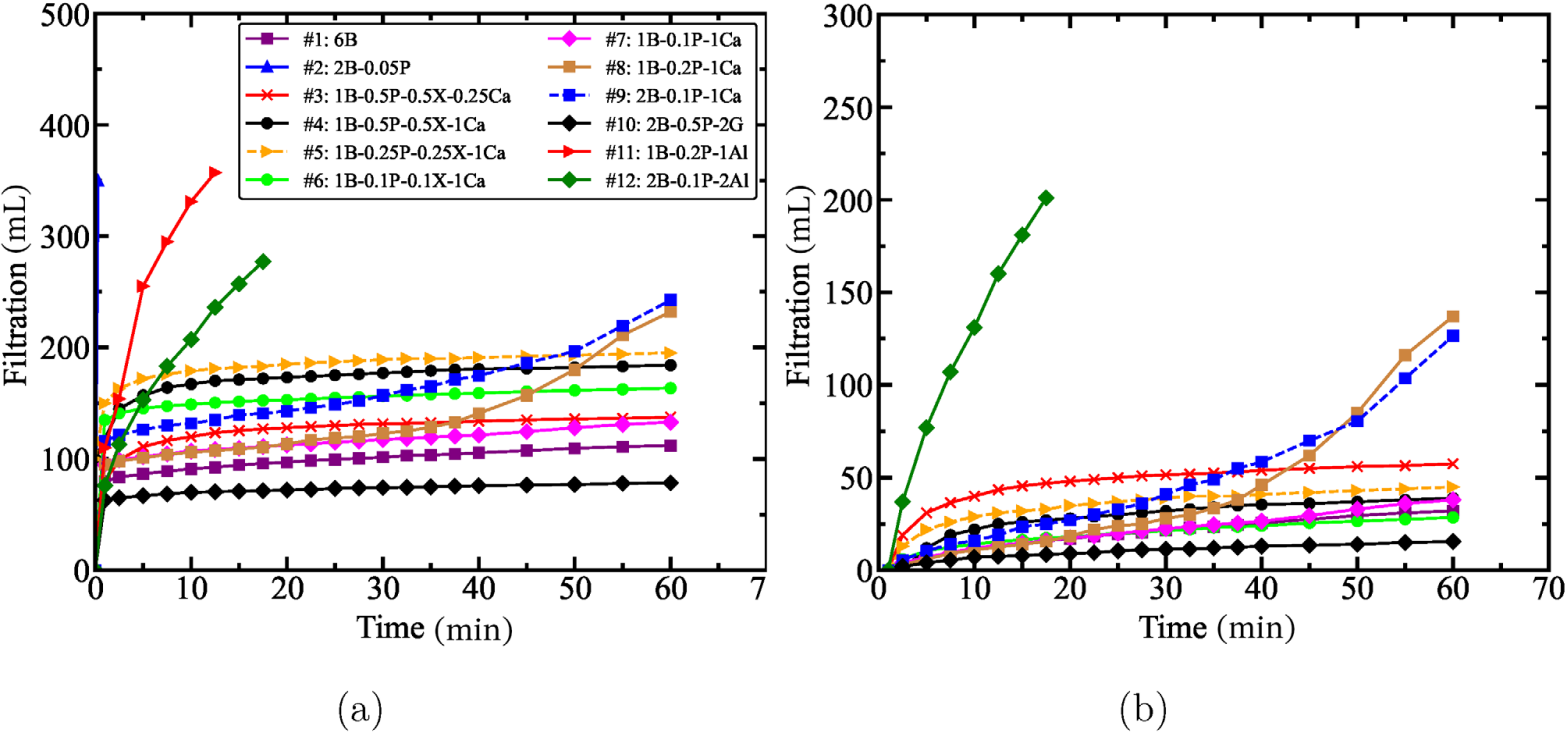
Cumulative filtration during the modified filtration test. (a) Including the initial discharge; (b) excluding the initial discharge.

Figure 10 presents the filtration results from the modified filtration test, with Figure 10a showing the results from the onset of the application of pressure, whereas Figure 10b showing the results excluding the initial discharge, that is, after the filter cake starts to form. The main difference from the filter paper experiments is the pore size, which is here larger than the filter paper pore throat size, which significantly enhances the permeability of the soil sample. A conventional drilling fluid (drilling fluid #2) that does not contain bridging particles shows an instantaneous discharge of approximately 350 mL in the first 30 s. Even though the same drilling fluid performed excellently in the API filter press, that is, low filtration rate and small filter cake, it appears to be unsuitable for bridging the target formation given the pore throat size and corresponding hydraulic conductivity. The remaining drilling fluids have a similar initial discharge and variations may be due to differences in the initial sand column height (ranging from 6.5 to 7.5 cm) as well as differences in the drilling fluid composition.

Drilling fluids #11 and #12, which contain the aluminum chloride hexahydrate as a bridging additive, perform poorly on reducing the permeability and exhibited a high filtration rate. All other fluids significantly reduce the filtration rate, even at relatively low concentrations.

**Figure 11:**
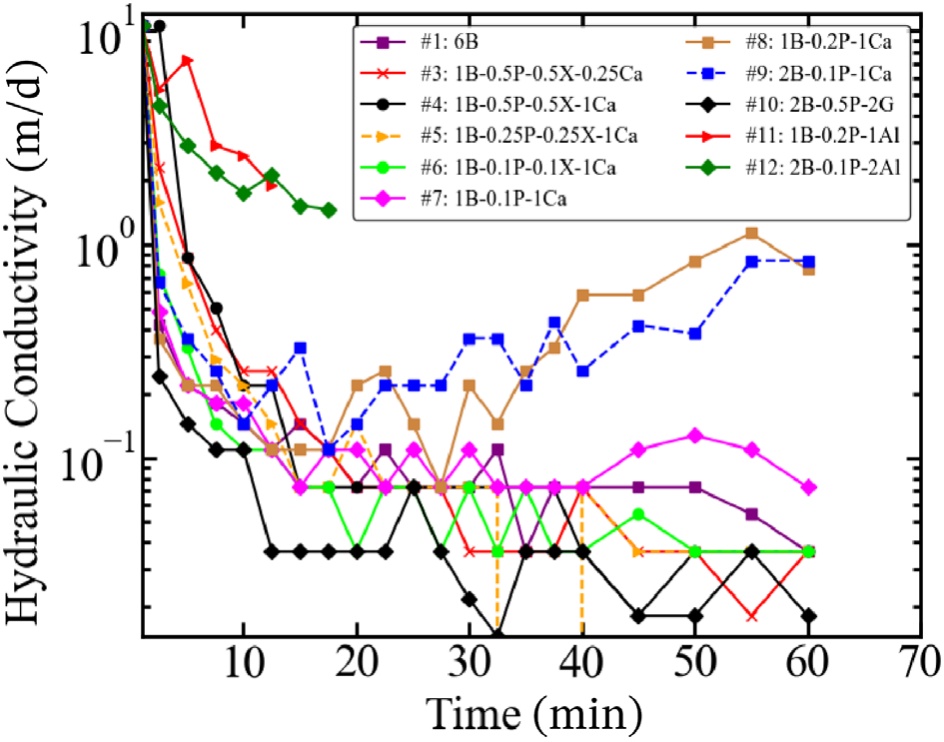
Calculated hydraulic conductivity during the filtration tests. The hydraulic conductivity of the samples shows an exponential decrease while conducting filtration tests.

By reducing the concentration of CaCO_3_, slightly higher losses are observed, yet still within the acceptable limit. Even though gypsum is not a common additive for wellbore bridging, the filtration tests show that gypsum provides the best sealing of the formation. Drilling fluids #8 and #9 show a significant reduction in filtration rates, similar to other drilling fluids, yet this is followed by an increase after about 30 min. On the other hand, this pattern is not observed in Figure 9a in comparison to drilling fluid #7. Figure 11 shows the effect of the tested drilling fluids on the hydraulic conductivity of the sample as a function of time. Compared with the hydraulic conductivity of the original sample, the hydraulic conductivity of drilling fluid #10 during testing shows the largest decrease, which is 99.8%.

### SEM Images

**Figure 12:**
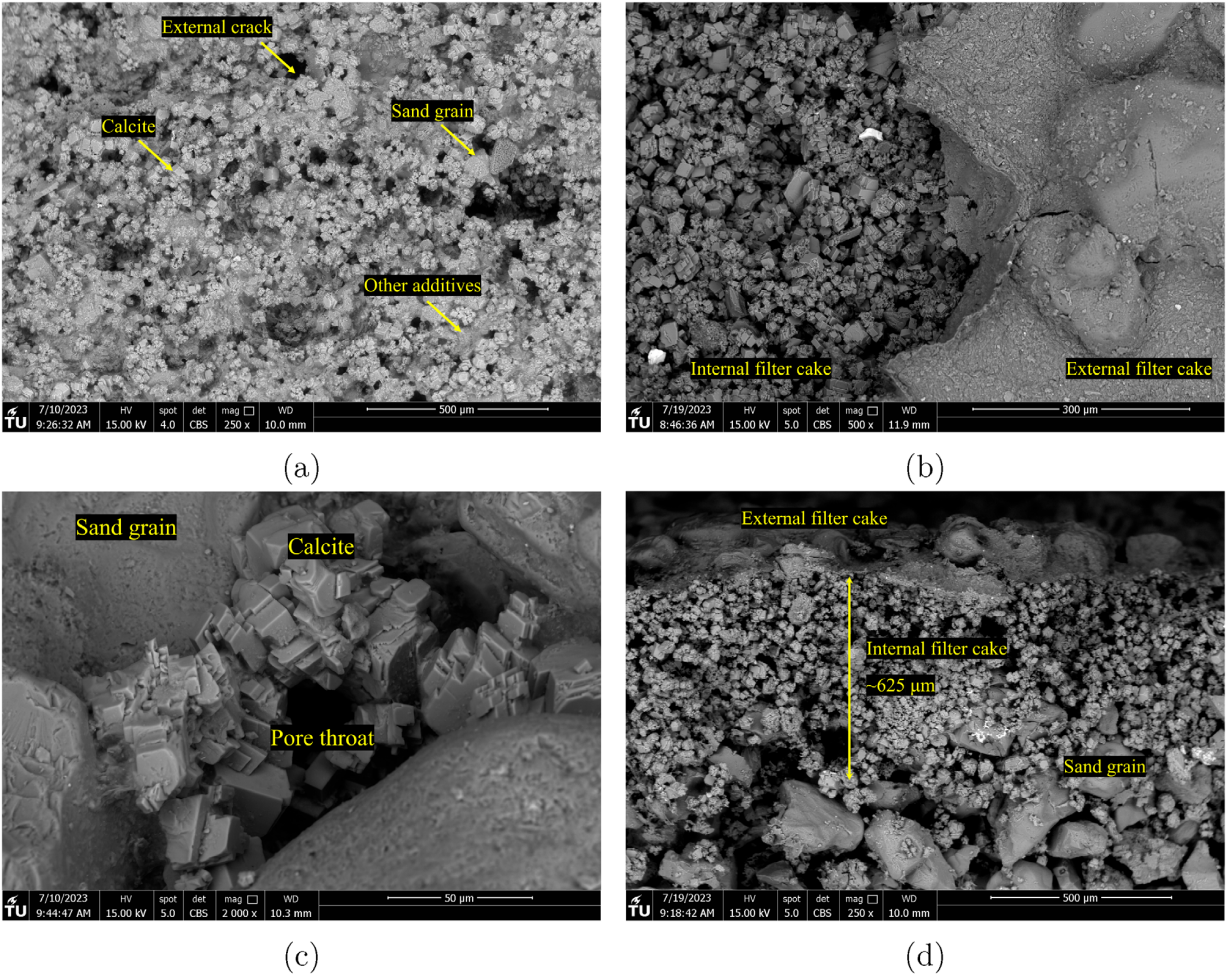
SEM images of the filter cakes formed. (a) Top view of the filter cake formed using drilling fluid #6; (b) Top view of the filter cake formed using drilling fluid #8; (c) Calcite crystals bridging the pores of the formation using drilling fluid #6; (d) External and internal filter cake formed using drilling fluid #8.

**Figure 13:**
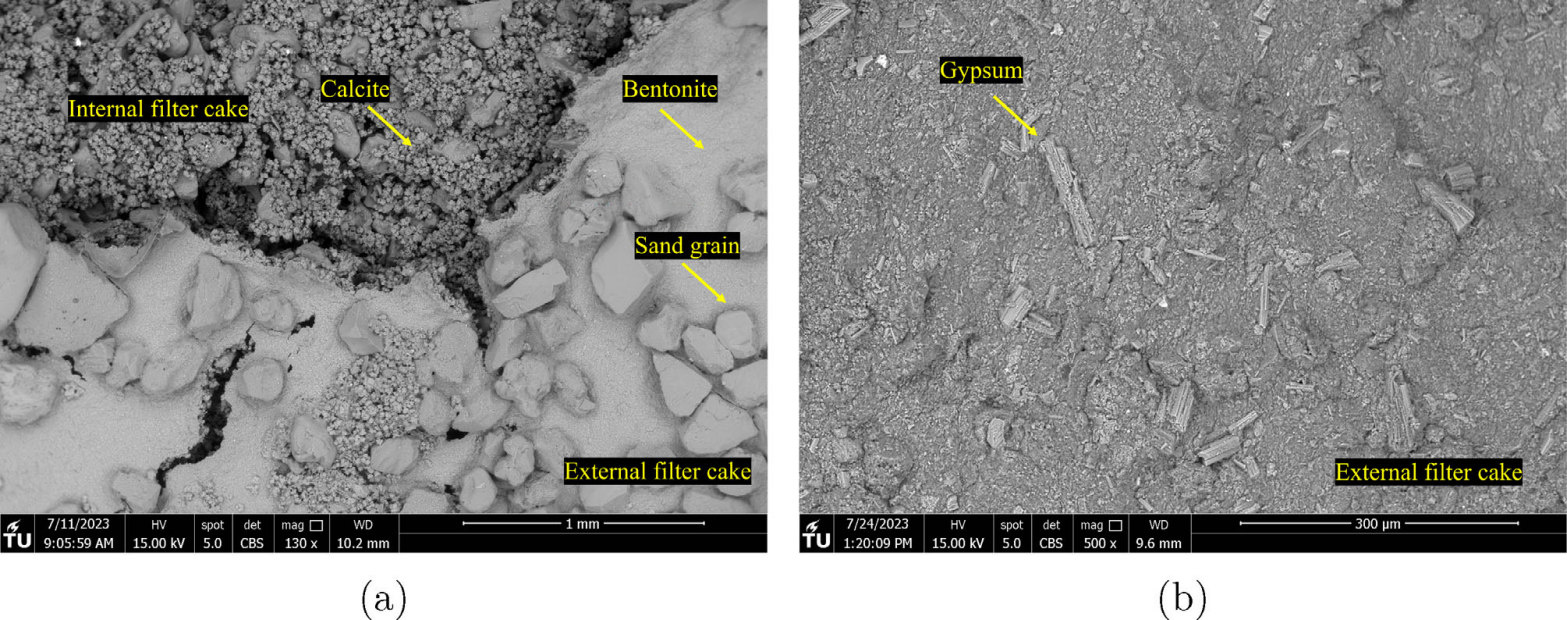
Top views of SEM images (a) A representation of the surface of the internal and external filter cake for drilling fluid #7; (b) Formed filter cake from drilling fluid #10.

Figures 12 and 13 show a selection of the SEM images, with Figure 12 showing the top view and side views of filter cakes produced from drilling fluid #6 and #8, respectively. It is observed that calcium carbonate effectively bridges the pores of the formation. Figure 13 shows the different filter cake surfaces from drilling fluids #7 and #10. A closer examination of the top view of the identified interval filter cake produced from drilling fluid #7 (Figure 13a), shows a further penetration of the calcite crystals in the formation since sand grains are considerably more evident compared to the top view of the internal filter cake for drilling fluid #8 (Figure 12b). Gypsum particles show an effective filtration control by forming an impermeable filter cake illustrated in Figure 13b.

The external filter cake structure and morphology are a preliminary indication of the filtration process. The growth of the internal and external filter cake is evidenced in Figure 12d, with primarily calcite particles at the internal and small particles at the external. In addition, the homogenous thickness of the external filter cake demonstrates the gradual suspension of the heavier calcite and lighter bentonite particles and the uniform filtration across the sample. The main difference between drilling fluid #6 and #8 is the concentration of the Xanthan Gum. We observe from the rheological experiments that Xanthan Gum significantly increases the viscosity of the prepared fluid and thus affects the particles settling velocity of particles (reversely proportional to viscosity), as is illustrated in Figure 12a and 12b. It was expected that the greater the percentage of the invaded CaCO_3_ particles in the formation, the lower the filtration losses. The filtration data (Figure 10) show that during the filtration experiments with drilling fluid #8, has more significant losses than drilling fluid #6. The formed filter cake from drilling fluid #6 shows a significant concentration of calcium carbonate on the surface. The side view of the tested sand samples in Figure 12d shows that the interval filter cake's penetration depth is uniform when injecting the drilling fluid #8 with a distinct separation from the rest of the sand sample. During the experiment with drilling fluid #8, calcium carbonate particles are observed even 650 *μ*m from the surface of the filter cake.

**Figure 14:**
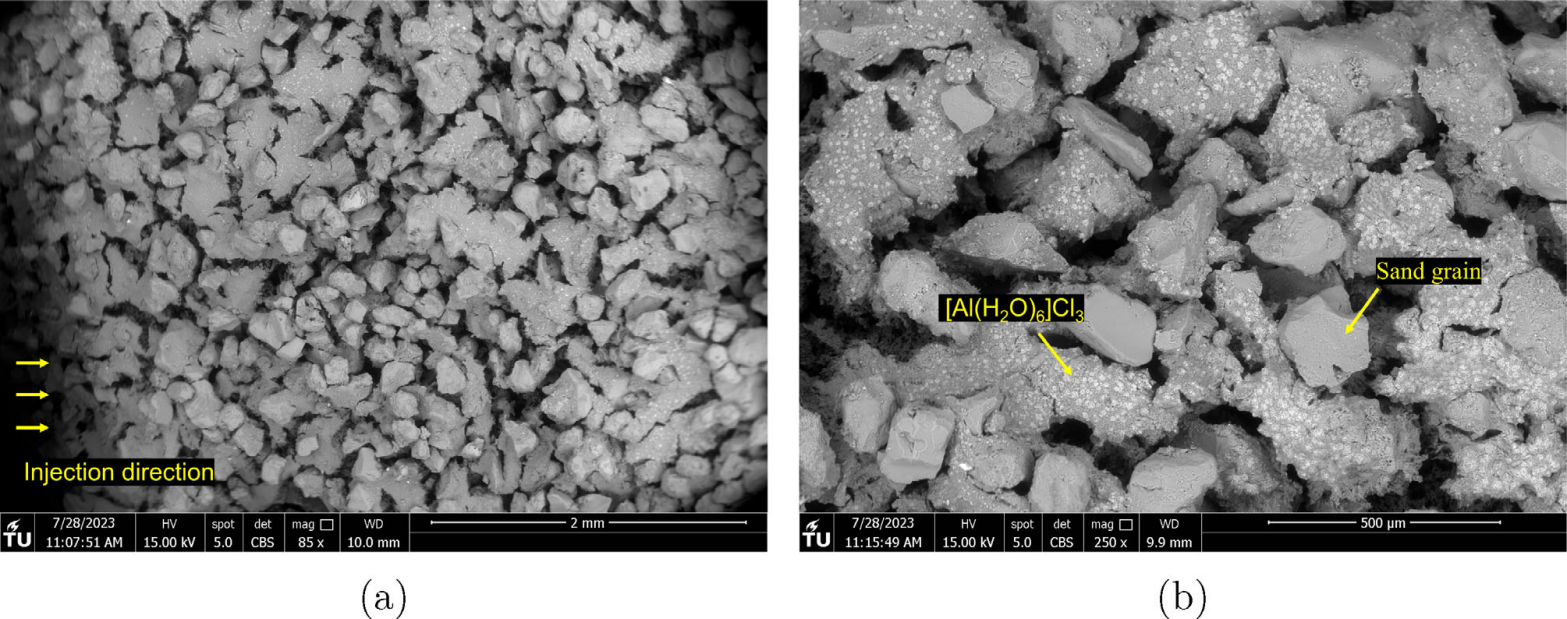
SEM images of the internal filter cake formed using drilling fluid #12 (a) Filter cake extent; (b) Details of the bridging mechanism.

The internal filter cake is further investigated using SEM. The filter cake produced from drilling fluid #12 has a thickness of approximately 4 mm (Figure 14a). Figure 14b explores the details of the filter cake. While the aluminum chloride hexahydrate coats the sand grains, the pores are not bridged and a substantial flow volume remains. This confirms the hypothesis that while the filter cake thickness is important for both strengthening and flow restriction, details of the pore bridging mechanism are also important.

### Fall Cone Penetrometer

**Figure 15:**
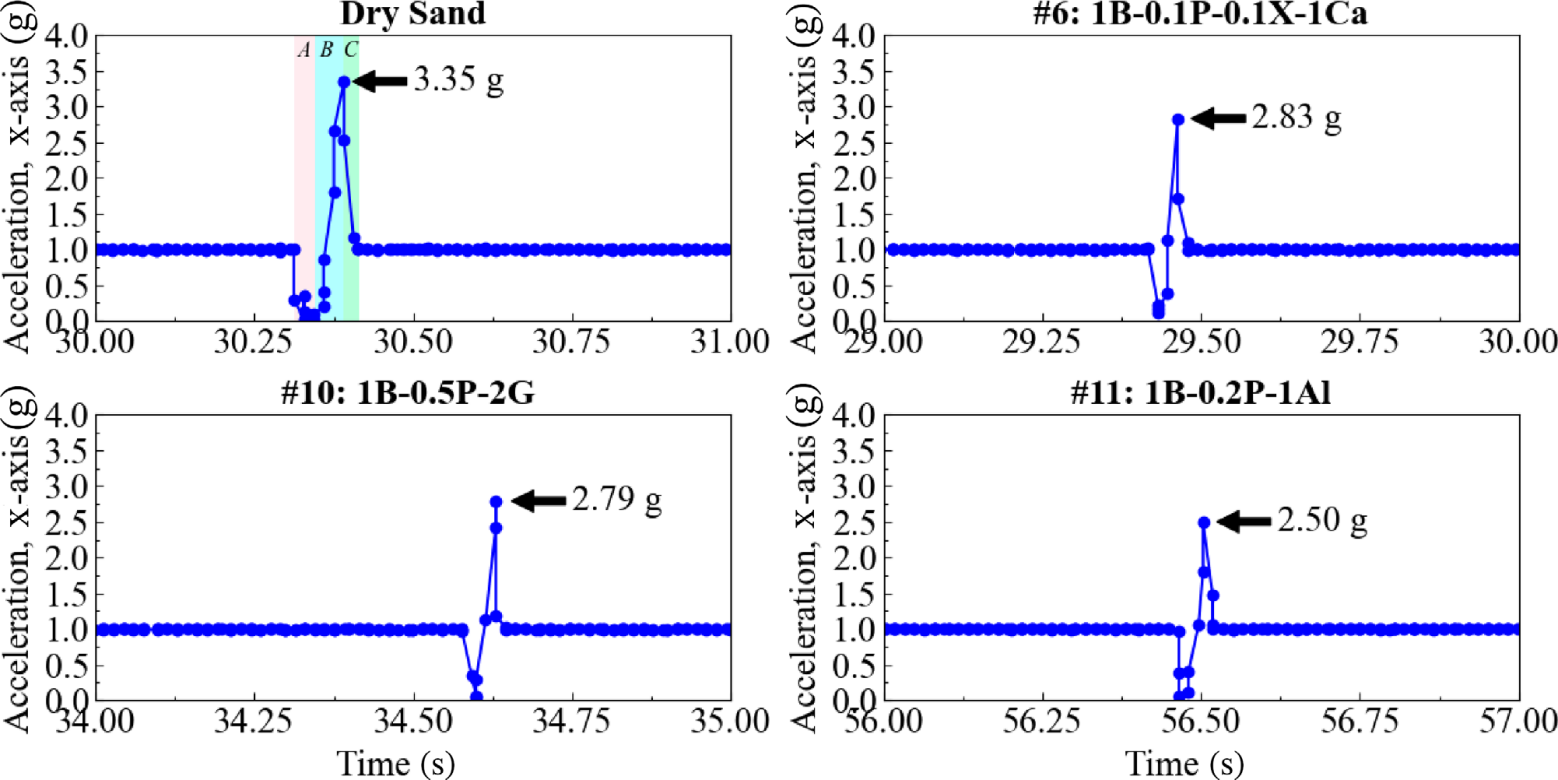
Selected penetration acceleration/deceleration measurements during the fall cone penetrometer test.

Figure 15 shows selected results from the accelerometer attached to the penetrometer. The data show the three distinct stages, that are indicated in Figure 6, highlighted on the top left sub‐figure.

In drilling fluids #6 and #10, the maximum deceleration is larger than for drilling fluid #11, indicating a greater impact on wellbore strengthening. In addition, the time of Stage B (*t*_*B*_), while penetrating the dry sample, shows a longer duration, corresponding to deeper penetration of the cone and therefore a weaker formation. The deceleration during cone penetration in the samples saturated with drilling fluid is lower in all cases than that in the dry sand experiment.

Table 3 presents key results from the fall cone penetrometer tests, SEM images, and modified filtration tests. The internal filter cake thickness (*h*) is not acquired from each of the samples due to the similar composition and behavior of some drilling fluids. For the tests performed in the unsaturated sand and with drilling fluid #2, *t*_*B*_ is significantly higher than the other tests, indicating a relatively low effect on wellbore strengthening. The interval filter cake thickness varies from 0.10 to 0.62 mm without a direct indication of relation with the filtration amount (and therefore hydraulic conductivity).

**Table 3:** Compilation of Test Parameters

Name	*t*_*B*_ (s)	*t*_*B*&*C*_ (s)	*d* (mm)	Max. decel. (g)	*h* (mm)	*q* (mL)
Dry sand	0.012280	0.01754	22.07	3.35	—	—
#1: 6B	0.007018	0.01052	6.67	3.15	—	9.0
#2: 2B‐0.05P	0.010526	0.01578	7.60	2.16	0.10	350
#3: 1B‐0.5P‐0.5X‐0.25Ca	0.007018	0.01228	8.02	2.69	—	36.5
#4: 1B‐0.5P‐0.5X‐1Ca	0.008772	0.01403	6.27	1.96	—	19
#5: 1B‐0.25P‐0.25X‐1Ca	0.007018	0.01228	7.21	2.79	—	26
#6: 1B‐0.1P‐0.1X‐1Ca	0.007018	0.01052	5.58	2.83	0.50	12.5
#7: 1B‐0.1P‐1Ca	0.007018	0.01228	6.62	2.44	0.25	9.5
#8: 1B‐0.2P‐1Ca	0.005263	0.00877	6.02	3.03	0.62	9.0
#10: 2B‐0.5P‐2G	0.008772	0.01228	6.20	2.79	0.30	5.5
#11: 1B‐0.1P‐1Al	0.008772	0.01228	6.12	2.50	—	185
#12: 2B‐0.1P‐2Al	0.008750	0.01225	6.43	2.62	4.00	107

Notes: *t*_*B*_, *t*_*B*&*C*_, penetration depth *d*, and the maximum deceleration are from the fall cone penetrometer results, the internal filter cake thickness *h* is determined from the SEM images (where no data is presented, SEM images were not acquired for that sample), and the filtration amount within 7.5 min *q* is from the modified filtration test. For drilling fluid #9 the core was disturbed, thus no tests were performed.

**Figure 16:**
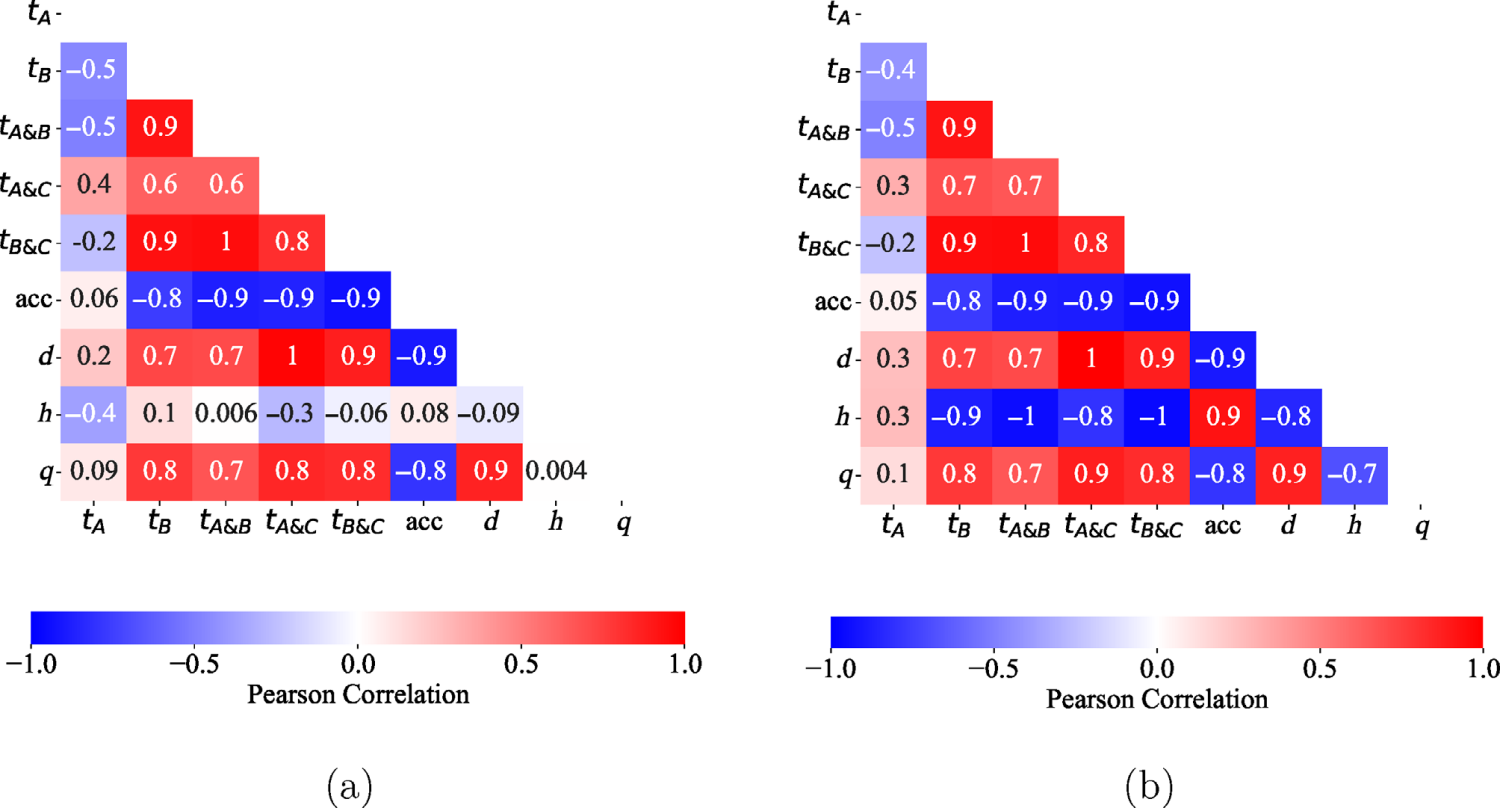
Pearson correlation matrix of the experimental results. (a) Considers drilling fluids #2, #6, #7, #8, #10, and #12; (b) Excludes drilling fluid #12.

The relationship (correlation) between the variables presented in Table 3 is investigated using the Pearson correlation coefficient. Figure 16a presents the Pearson correlation coefficients between the different data collected, that is: the time taken in the stages of the fall cone test, the deceleration (acc), the cone penetration depth (*d*), the internal filter cake thickness (*h*), and the filtration loss (*q*) for the initial 7.5 min of the modified filtration test, for the drilling fluids with all data available (drilling fluids #2, #6, #7, #8, #10, and #12). In Figure 16b, drilling fluid #12 is not included, since Table 3 shows that drilling fluid #12 forms a thick but permeable filter cake.

In Figure 16a, as expected, the cone penetration depth is inversely correlated to the filter cake thickness; thus, the deeper the filter cake penetrates the formation, the lesser the cone penetration in the sample. However, this correlation is weak. It can also be observed that the deceleration is strongly inverse‐correlated with *t*_*B*_, *t*_*A*&*B*_, and *t*_*A*&*C*_ (note *t*_*A*&*C*_ is the time for Stages A, B, and C). This indicates that the longer the time taken to accelerate and decelerate, the smaller the maximum deceleration. In addition, there is a strong negative correlation between the maximum deceleration and penetration depth, meaning that low penetration depths have high decelerations indicating a stronger sample, and a strong positive correlation between penetration depth and *t*_*B*_, *t*_*A*&*B*_, and *t*_*A*&*C*_. From a physical perspective, this indicates that wellbore strengthening occurs due to drilling fluid components bridging pore throats and providing additional cohesion forces between the particles and thus enhances the shear strength of the soil.

The filtration losses are seen to be positively correlated with penetration depth, that is, positively correlated with a stronger sample, and inversely correlated with the deceleration. While the filtration losses are also impacted by the external filter cake, it is also concluded that the internal clogging/bridging of pores inside the formation plays an important role. However, the internal filter cake thickness is not well correlated with any of the tested parameters. This is hypothesized to be due to the drilling fluids containing aluminum chloride hexahydrate forming a thick internal and external filter cake, which is ineffective in impeding flow. It is seen to have a moderate wellbore strengthening effect (the fall cone penetrated 6.43 mm). Figure 16b provides the same analysis, excluding drilling fluid #12. Internal filter cake depth is strongly correlated to deceleration, and strongly inversely correlated to the penetration depth, filtration losses and times *t*_*B*_, *t*_*A*&*B*_, and *t*_*A*&*C*_. This implies that when a drilling fluid is generally performing well, the thickness is a good proxy for overall performance, but if the mechanisms that cause the filter cake do not meet one of the required functions it cannot. Therefore, both the cone penetration and flow aspects of the test are needed. As expected, the *t*_*A*_ is not well correlated to any of the other results, as this is related to the initial acceleration and should not be related to filter cake or formation. This is an indication that the experiment is performing as expected.

## Discussion

### Effect on Filtration Losses and Wellbore Strengthening

Drilling fluid #1 does not contain any additive that is specifically designed to assist in pore throat bridging, but we can still observe a significant reduction in filtration losses, the second lowest value in the experimental campaign, indicating a good performance of reducing losses. The fall cone results show a high maximum deceleration and relatively small cone penetration depth, which corresponds to the impact of the bridging additives on wellbore strengthening. However, due to the high amount of bentonite, the high resulting fluid viscosity means it is practically difficult to drill with. The conventional drilling fluid #2 shows an instantaneous discharge of all drilling fluid in the modified filtration test, despite showing reasonable performance on the API filter press, which is related to the fact that a filter cake is not formed as the pores are not bridged. This indicates poor fluid control, which would result in substantial fluid losses and elevated pore pressures in the field, which could result in wellbore instability or collapse. In addition, there is a large penetration depth, indicating a low strengthening effect due to the small internal filter cake. The results indicate this reduction in the filtration rate and enhancement of wellbore strengthening, it is not only a function of filter cake thickness but also the bridging mechanism of the additives in the pores.

**FIGURE 17:**
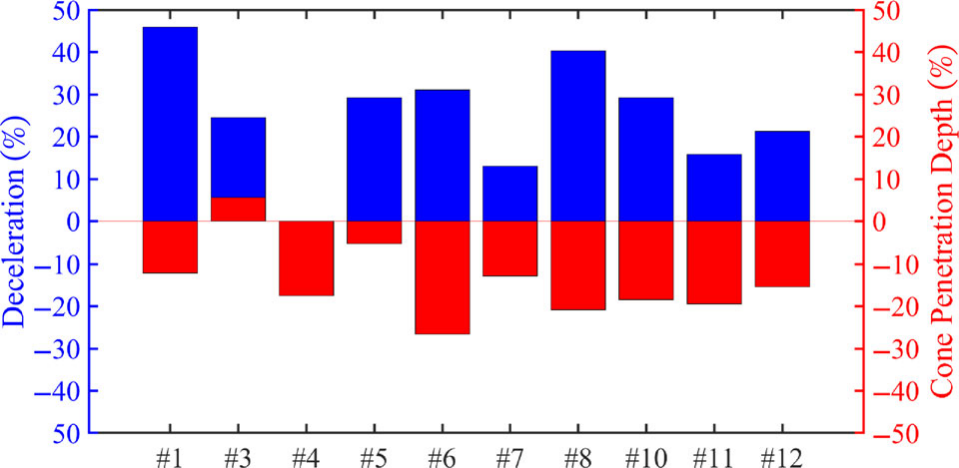
A representation of the increase in deceleration and decrease of cone penetration depth with respect to the obtained values of drilling fluid #2.

Drilling fluid #10 has an excellent capability to reduce the filtration rate, without forming an internal filter cake. The deceleration is increased by 29% and the penetration depth of the cone is decreased by 18.5% (Figure 17), indicating that the internal filter cake is strong even though it is very small.

Even though drilling fluids #3 and #4 have low filtration rates, the corresponding increase of plastic viscosity and gel strength are limiting factors, and therefore, these drilling fluids are not suited to be used in combination with reverse circulation drilling. Calcite particles play a filling role in bridging the formation, with the performance increasing with the additional of PAC and Xanthan Gum, which act as viscocifiers and assist in reducing the setting velocity of the particles. The modified filtration tests demonstrate that by removing Xantham gum as an additive and slightly increasing the concentration of PAC or bentonite, drilling fluids #8 and #9 show a corresponding increase in the filtration rate. The calcite crystals bridge the pore throat and thus reduce the permeability in the near wellbore region. The fall cone penetration depth decreases significantly, indicating an increase in strength (Figure 17).

The aluminum chloride hexahydrate (drilling fluids #11 and #12) shows no bridging effect for the expected pore throat size. Previous studies have shown that aluminum‐based drilling fluids were excellent stabilizers for drilling shale intervals (Ramirez et al. 2005; Buranaj Hoxha et al. 2022), but despite the formation of a thick internal filter cake, they remain highly permeable with no significant effect on wellbore strengthening.

### Field Application and Well Development

The methodology for conventional deep wells in unconsolidated formations is primarily to increase the wellbore strengthening and successfully drill the section without considering later well development practices. For shallow wells with the target in unconsolidated formations, the effect of the drilling fluid on wellbore strengthening and well development practices needs to be considered. Thus, the selection of the optimum drilling fluid needs to consider wellbore strengthening, rig and drilling limitations, environmental impact (as these formations are often used for drinking water extraction), and well development practices. Average pore throat size is a fundamental variable that assists in selecting the optimum size of the bridging particles.

**Figure 18:**
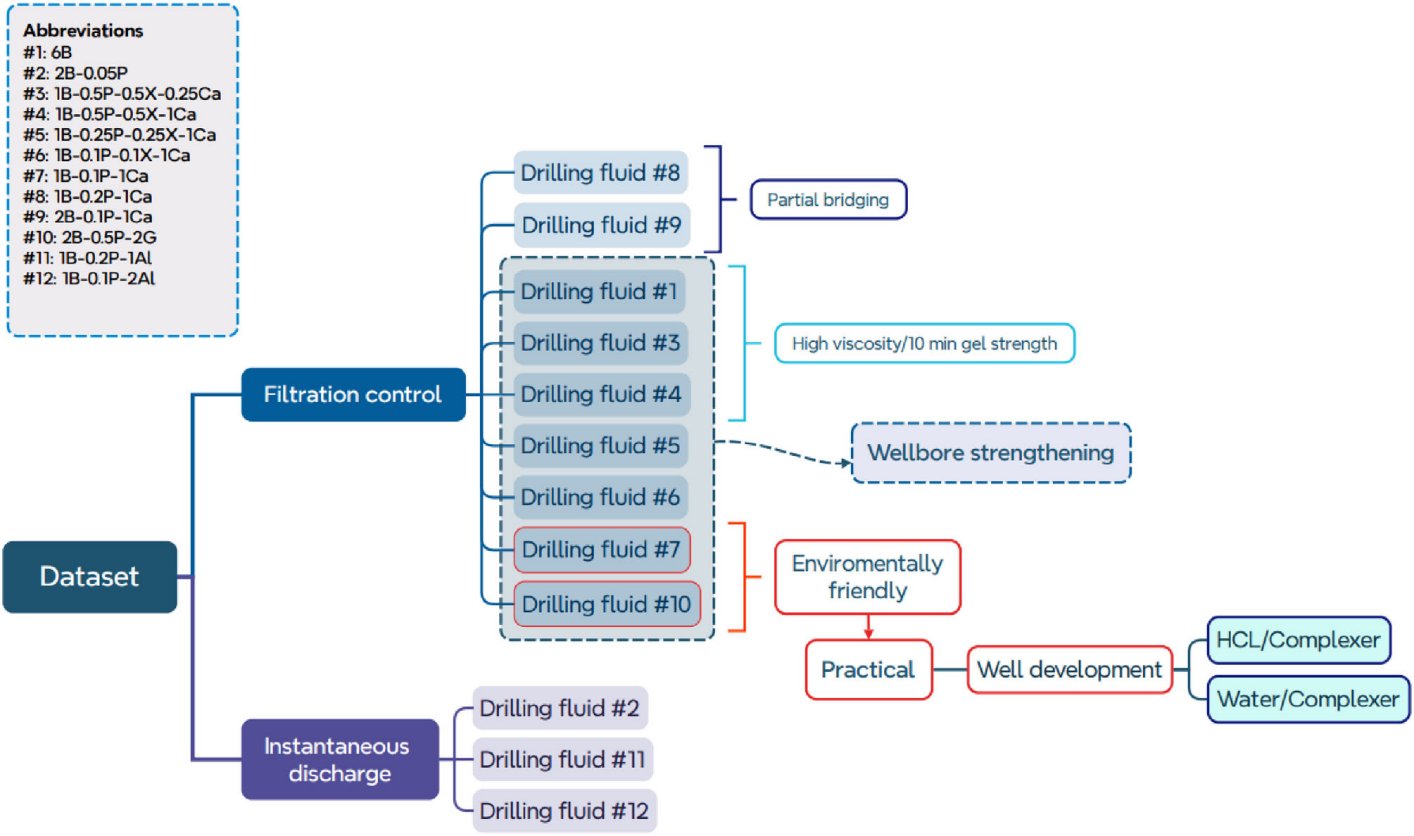
General overview of the obtained dataset and analysis, considering the practical aspects and well development practices. A complexer is an active substance that removes specific additives from the drilling fluid.

The following Figure 18 provides an overview of the entire dataset and analysis, considering the practical aspect of drilling rig mechanical limitations and environmental regulations. Drilling fluids #2, #11, and #12 are seen to have poor pore throat bridging and thus exhibited instantaneous discharge and are therefore not suitable. As was mentioned, filtration control does not necessarily correspond to an increase in wellbore strengthening, and the tested fluids perform differently in the API filter press and the modified apparatus. Most of the drilling fluids show a rapid control of fluid loss into the formation. A slight modification of the concentration of PAC and bentonite shows that after approximately 30 min, drilling fluids #8 and #9 perform poorly, and therefore are also disregarded.

Drilling rig mechanical limitations are associated with the maximum operational plastic viscosity limit of the drilling fluid that can be used during reverse circulation. The plastic viscosity of drilling fluids #3 and #4 are significantly higher than the benchmark value of 9 cP. On the contrary, drilling fluid #1 shows permissible plastic viscosity, but due to the high concentration of bentonite, it produces a high 10 min gel strength. Therefore, these drilling fluids are not further considered.

Unlike deep wells, shallow wells are primarily drilled with a single‐size drill bit to the target depth. Thus, the shallow fresh groundwater is exposed to the drilling additives while drilling to and through the target formation. PAC has a lower bacterial growth potential than Xanthan Gum and thus, it is preferred in groundwater wells (Timmer and Pittens 2007). Xanthan Gum is associated with bacteria growth, which increases the effect of biological clogging and elevated microbial activity above drinking water standards. Drilling fluids #5 and #6 provide optimal rheological properties and effective pore throat bridging but are limited to applications which do not include drinking water extraction.

The filter cake is a cohesive elastoplastic material, in which if the applied stress is greater than the shear strength, it will flow and thus cause its removal. In deep wells, in order to ensure good bonding between the casing‐cement‐formation, a scratching device (bottlebrush) is lowered in the wellbore to remove the filter cake that remained after the drilling process. The main physical element that provides strength in the filter cake is the cohesive forces between the solid particles. Due to the high hydraulic conductivity and large pore throat size, the internal filter cake is up to double the size of the external. Thus, chemical well development is the primary solution to remove those particles. In the case of drilling fluid #7, a common operation to regain the formation's hydraulic conductivity is by injecting hydrochloric acid (HCL), which reacts and dissolves the calcium carbonate particles. Regarding drilling fluid #10, for applications where the drilling fluid is not exposed to high temperature, gypsum is soluble in water, and thus, the formulated filter cake can be easily removed.

## Conclusions

An experimental framework is designed and implemented to test drilling fluids under typical downhole conditions for wells in shallow unconsolidated aquifers. Such drilling operations often use drilling fluids with limited strengthening properties, and these drilling fluid usually come in contact with drinking water aquifers due to single‐string well designs. This leads to a different set of requirements than in deep drilling projects. Various aspects are investigated, including filtration losses, penetration depth of the internal filter cake, and wellbore strengthening. This study provides supportive evidence for selecting well‐performing drilling fluids for unconsolidated formations. The methodology from selecting the optimum bridging particle size to experimental validation is not limited to this particular formation, but it can be applied to unconsolidated formations with different physical properties. Aggregate data obtained from the modified apparatus, fall cone penetration and SEM can reveal and effectively assess the wellbore strengthening. Salient conclusions on the drilling fluids tested are:

- The conventional drilling fluid design shows excessive filtration rates corresponding to low wellbore stability.
- Calcite crystals in the drilling fluid cause an almost instantaneous bridging of the pore throats, resulting in reduced filtration loss. Even though bentonite particles mainly form an external filter cake, most of the particles that bridge pore throats in the formation are calcite crystals.
- The [Al(H_2_O)_6_]Cl_3_ demonstrates a poor bridging mechanism and thick internal and external filter cake.
- Viscofiers (PAC and Xanthan Gum) change the settling velocity of calcite crystals which impacts the composition of the external and internal filter cake.
- CaCO_3_ particles result in a decrease of cone penetration depths of up to 20.78% and a 40.27% increase in deceleration while penetrating the sample compared to conventional drilling fluid containing bentonite and PAC, indicating enhanced wellbore stability.
- Even though gypsum is not a common filtration agent, for the tested sand formation, it forms an impermeable thin filter cake that significantly reduces fluid losses and enhances wellbore strength.
- Drilling fluids that contain calcite crystals or gypsum, low PAC concentration, and without Xanthan Gum, comply with the mechanical limitations of typical shallow drilling rigs, environmental regulations, and available well development practices.

## Authors' Note

The authors do not have any conflicts of interest or financial disclosures to report.

**Notation**
acc: deceleration (g)
*d*
: cone penetration depth (mm)
*h*
: internal filter cake thickness (mm)
*μ*_*p*_
: plastic viscosity (cP)
*y*_*p*_
: yield point (lb/100 ft^2^)
*d*_*p*_
: particle diameter (*μ*m)
*r*_pore_
: pore radius (*μ*m)
*t*
: time of a stage during cone penetration (s)

## Data Availability

The data that support the findings of this study are available from the corresponding author upon reasonable request.
